# Expression Patterns of miR‐29b and miR‐148a in Colostrum of Awassi Sheep With Higher Immunoglobulin G Levels

**DOI:** 10.1002/vms3.70335

**Published:** 2025-04-04

**Authors:** Hüseyin Özkan, Ufuk Kaya, Hasan Hüseyin Keçeli, Ramazan Sertkol, Mustafa Kemal Saribay

**Affiliations:** ^1^ Department of Genetics Faculty of Veterinary Medicine Hatay Mustafa Kemal University Antakya Hatay Turkiye; ^2^ Department of Biostatistics, Faculty of Veterinary Medicine Hatay Mustafa Kemal University Antakya Hatay Turkiye; ^3^ Department of Veterinary Obstetrics, Institute of Health Sciences Gynecology, and Artificial Insemination Hatay Mustafa Kemal University Antakya Hatay Turkiye; ^4^ Department of Obstetrics and Gynecology, Faculty of Veterinary Medicine Hatay Mustafa Kemal University Antakya Hatay Turkiye

**Keywords:** colostrum quality, Immunoglobulin G, miR‐148a, miR‐29b, sheep

## Abstract

This study aimed to investigate the compositional parameters and expression patterns of miR‐29b and miR‐148a in sheep colostrum containing low and high levels of Immunoglobulin G (IgG). Blood and colostrum samples were collected within the first 6 h following birth from 24 pregnant Awassi sheep. On the basis of the determined colostrum IgG levels, low (Low IgG [LIGG], 44.83 ± 4.97 mg/mL) and high (High IgG [HIGG], 80.71 ± 3.31 mg/mL) groups were formed. In addition to measuring colostrum compositional parameters, expressions of miR‐29b and miR‐148a were determined in plasma and colostrum. Although somatic cell score (SCS), pH and electrical conductivity (EC) were higher, fat‐free dry matter (FFDM), protein, lactose and freezing point were lower in HIGG group. Compared to LIGG, miR‐29b was downregulated approximately 5‐fold in the HIGG, whereas miR‐148a was downregulated more than 3‐fold in colostrum. There were negative correlations between colostrum miR‐148a and colostrum IgG, SCS and pH. Colostrum IgG was positively correlated with pH and freezing point and negatively correlated with FFDM, protein and lactose. Area under the curve (AUC) values of SCS, pH, FFDM, protein, lactose, freezing point, EC and miR‐148a (colostrum) were significant. Sensitivity and specificity of pH were 100% and 66.7%, of SCS were 81.8% and 66.7% and of miR‐148a (Colostrum) were 75% and 75% in HIGG and LIGG. According to the results, miR‐29b might be an important molecular target for lamb development, whereas miR‐148a could be a potential biomarker for colostrum quality. Moreover, the study showed that SCS and pH might be useful diagnostic parameters for IgG status because of higher sensitivity rates and AUC values.

## Graphical abstract

miR‐29b might be an important molecular target for lamb development, whereas miR‐148a could be a potential biomarker for colostrum quality. SCS and pH might be useful diagnostic parameters for IgG status.

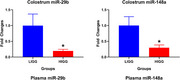

## 1 Introduction

Sheep, among the earliest domesticated farm animals, have been a crucial source of essential animal products for thousands of years. Sheep farming is particularly significant in developing countries as an alternative source for meat, milk and dairy products (Kasapidou et al. 2021; Simões et al. 2021). With the increasing global population, the demand for animal products is continuously rising, leading to higher production targets. It is projected that by 2050, global meat production will increase by approximately 20%, and sheep meat production will rise by around 4 Mt (Simões et al. 2021). The economic viability of sheep farming, their ability to better utilize poor pastures compared to cattle, the gestation period and the number of offspring per litter make sheep farming particularly prominent (Banchero et al. 2015; Simões et al. 2021).

All mammals require colostrum immediately after birth to access essential nutrients and immunity factors for survival. Newborns need to rapidly and promptly obtain immunity‐related molecules like Immunoglobulin G (IgG) found in colostrum (Banchero et al. 2015). The presence of immunity‐related factors and compositional parameters plays a significant role in determining colostrum quality. The time taken to access colostrum and its content are crucial for the survival and health of the offspring. Due to the structure of the placenta in ruminants, lambs are born agammaglobulinemic. The placental structure prevents the transfer of maternal immunoglobulins to the foetus, so lambs acquire their initial immunity through colostrum after birth. During the late gestation period, through the process of colostrogenesis, maternal immunoglobulins transfer from maternal blood circulation to the mammary gland. The amount of IgG is known to be the primary parameter in determining colostrum quality. Consequently, colostrum with high IgG (HIGG) content is considered to have high quality (Kessler et al. 2019).

Colostrum IgG concentrations are influenced by environmental factors as well as genetic factors such as breed. Although extensive information is available on colostrum IgG concentrations in cattle and goats, studies on sheep colostrum IgG levels are limited (Kessler et al. 2019; Pérez‐Marín et al. 2023). Additionally, knowledge about the relationships between IgG content and the compositional parameters of colostrum is also limited. Although radial immunodiffusion (RID) is gold standard, transmission infrared (IR) spectroscopy, digital and optical Brix refractometers and the ELISA methods are generally used to determine colostrum quality. It is known that most of these methods have limitations in terms of feasibility, accuracy and validity (Alves et al. 2015; Costa et al. 2021; Elsohaby et al. 2017). Recent molecular studies have highlighted microRNAs (miRNAs) as biomarkers for productivity, health and quality. These short non‐coding RNA molecules act as post‐transcriptional regulators, influenced by epigenetic factors, and their levels vary in tissues and biological fluids under different physiological and pathological conditions (Van Hese et al. 2020; Yun et al. 2021; Özkan et al. 2024).

In this study, the levels of miR‐29b and miR‐148a, which are reported to be potentially related to the immune system and milk quality and are among the most abundant miRNAs in milk, were investigated in colostrum with low and high IgG content and in the plasma of the animals (Van Hese et al. 2020; Petracci et al. 2023). Additionally, in groups formed on the basis of colostrum IgG content, the levels of somatic cell score (SCS) and compositional parameters, such as pH, fat, protein and lactose, were determined, and the relationships between these parameters and miRNAs were examined.

## 2 Materials and Methods

This study was conducted in Reyhanlı district, affiliated with Hatay province in the Eastern Mediterranean region of Turkiye (Latitude: 36° 15′ N; Longitude 36° 34′ E). Twenty‐four nulliparous sheep of the Awassi breed were impregnated using routine synchronization protocol (with flugestone acetate, PMSG and d‐cloprostenol). During pregnancy, in addition to grazing in the pasture, each sheep was given a concentrated feed containing 2700 kcal/kg energy and 16% crude protein daily, ranging from 500 to 600 g. Within the first 6 h postpartum, blood and colostrum samples were collected under sterile conditions. Blood samples were collected from the Jugular Vein into 9 mL EDTA tubes under sterile conditions, whereas colostrum samples were collected after ensuring mammary asepsis, with the first milking being discarded. All samples were transferred to nuclease‐free tubes and rapidly transported to the laboratory under cold chain conditions. Blood samples were centrifuged at 3000 × *g* for 10 min at +4°C to obtain plasma, which was stored at −80°C until miRNA and IgG analyses were conducted.

The collected colostrum samples were divided into two groups based on IgG levels, with an equal number of animals in each group (*n* = 12) designated as Low IgG (LIGG) and HIGG. The measured IgG levels in the colostrum of LIGG were 44.83 ± 4.97 mg/mL, whereas it was determined as 80.71 ± 3.31 mg/mL in HIGG.

### 2.1 Measurement of Compositional Colostrum Parameters

The colostrum samples were maintained at +4°C until the determination of somatic cell count, milk quality parameters and pH values. The analysis of somatic cell count was conducted using a Somatic Cell Count Device (Lactoscan SCC 6010, Bulgaria). Various milk quality parameters, including fat content, fat‐free dry matter (FFDM), protein, lactose, freezing point and electrical conductivity (EC), were measured using a Milkotester Device (Master Classic, M2, P1, Bulgaria). The pH values of the colostrum samples were determined using a pH meter (Hanna HI83141, USA). All measurements were carried out meticulously in accordance with the manufacturers’ instructions.

### 2.2 Determination of IgG Levels

IgG analysis from serum and colostrum samples was conducted using a sheep‐specific ELISA kit (ESH0060, FineTest, China) in accordance with kit protocols. Initially, an optimization was conducted to determine the appropriate dilution ratios, after which serum and colostrum samples were diluted at a 1/2 ratio with the kit's dilution solution for analysis. The results were determined using an ELISA Reader (AMR‐100, Allsheng, China) at a wavelength of 450 nm.

### 2.3 RNA Isolation, cDNA Synthesis and RT‐qPCR

RNA isolation was performed using the modified Trizol method (Rio et al. 2010). For this purpose, colostrum and serum samples stored at −80°C were thawed by incubating at room temperature for 10 min. Thawed samples of 250 µL were then transferred to nuclease‐free tubes containing 1 mL of Nucleogene Tri Reagent (Cat. No.: NGE023, Nucleogene, Turkiye) and incubated for 10 min at room temperature. Following chloroform, isopropyl alcohol and ethanol steps, RNA pellets were allowed to air dry at room temperature for 10 min and then resuspended in 20 µL of nuclease‐free water (Özkan and Kerman 2020). The purity and concentration of the samples were assessed using a nucleic acid analyser (SMA‐1000, Meriton, China).

The isolated RNAs were diluted with nuclease‐free water to a concentration of 500 ng/µL. Following polyadenylation with Poly(A) polymerase (Cat. No.: e017, ABM, CANADA), cDNA synthesis was performed using the Onescript Plus cDNA Synthesis Kit (G236, ABM, Canada) (Özkan et al. 2024). For this purpose, samples were incubated at 55°C for 15 min, followed by 85°C for 5 min to synthesize cDNA. The resulting equal volumes of cDNA were then brought to a final volume of 200 µL by adding nuclease‐free water.

The expression levels of miR‐29b and miR‐148a were determined using the GoTaq qPCR Master Mix kit (A6001, Promega, USA) with qPCR (Rotor Gene Q, Qiagen, USA) and subjected to 50 cycles of amplification at 95°C for 10 min, 95°C for 15 s, 57°C–60°C for 60 s and 72°C for 30 s. All samples were studied in duplicate, and SnoU6 was used as housekeeping for normalization. The primer sequences of the miRNAs used in the study are presented in Table 1. In addition, Universal 3′ miRNA Reverse Primer (MPH00000, ABM, Canada) was used as the reverse primer (Özkan et al. 2024).

**TABLE 1:** Primer sequences of the miRNAs and SnoU6.

miRNA	Primer sequences	Reference
Oar‐miR‐29b	5′‐TAGCACCATTTGAAATCAGTGT‐3′	miRBASE
Oar‐miR‐148a	5′‐TCAGTGCACTACAGAACTTTGT‐3′	miRBASE
SnoU6	F: 5′‐CTCGCTTCGGCAGCACA‐3′	Guo et al. (2018)
	R: 5′‐AACGCTTCACGAATTTGCGT‐3′	

### 2.4 miRNA Target Gene Prediction and Protein–Protein Interaction (PPI) Analysis

Human homologue miRNAs were utilized to predict the target genes of sheep miRNAs. This approach is supported by the extensive bioinformatic analyses and experimental studies available on human miRNAs, in contrast to the limited data concerning sheep miRNAs (Duman et al. 2024; Manenti et al. 2024). Furthermore, the high degree of conservation and functional similarity of miRNAs across species underscores the reliability of using homologous miRNAs in comparative analyses (Yun et al. 2021). For this purpose, the target genes of differentially expressed miRNAs (DEMs) were identified and visualized using the miRNet Centric Network Visual Analytics Platform (version 2.0, https://www.mirnet.ca/) with the miRTarBase database (Chang et al. 2020). Additionally, the number of target genes was visualized through a Venn diagram generated using the online tool jVenn (https://jvenn.toulouse.inrae.fr/app/index.html). After identifying the common genes targeted by the DEMs, PPI analysis was performed using the STRING database (https://string‐db.org/). In these analyses, the interaction score was set to >0.150, with a maximum interaction limit of 10 and 50 interactions for the 1st and 2nd shells, respectively (Szklarczyk et al. 2025). The PPI networks obtained from the STRING database were visualized using the STRING app in Cytoscape (version 3.10.3). High‐density clusters in the resulting networks were further analysed using the Molecular Complex Detection (MCODE) app. The default parameters for MCODE analysis (Degree Cutoff: 2, Node Score Cutoff: 0.2, K‐Core: 2, Max. Depth: 100) were applied (Zhang et al. 2022).

### 2.5 Functional Enrichment Analysis of DEM‐Regulated Target Genes

The Gene Set Enrichment Analysis (GSEA) of the common genes regulated by the DEMs was performed using the STRING database, and the Gene Ontology (GO) database was used to evaluate biological process (BP), cellular component (CC) and molecular function (MF) categories (Szklarczyk et al. 2023). Additionally, the roles of the target genes in biological pathways were analysed using the Kyoto Encyclopedia of Genes and Genomes (KEGG) and Reactome Pathways (Reactome) databases. In these analyses, results with a false discovery rate (FDR) of <0.05 were considered statistically significant (Benjamini and Hochberg 1995). Among the significant results obtained from the GO, KEGG and Reactome Pathways databases, the 15 parameters with those most consistent with the hypothesis highlighted were visualized using R software (version 4.4.2) and the ggplot2 package (version 3.5.1).

### 2.6 Statistical Analysis

Statistical analyses were performed using Stata SE version 15.1 and MedCalc Version 9.2. Variables were evaluated in terms of parametric test assumptions according to normality (D'Agostino‐Pearson Omnibus test) and homogeneity of variances (Levene test). SCC data were normalized and transformed to SCS (SCS = log2 (SCC/100.000) + 3) (Ali and Shook 1980). Descriptive statistics were calculated as ‘Mean ± Standard Error of Mean (SEM)’ and presented as figures. The differences in IgG (colostrum and plasma) and compositional parameters in colostrum among the groups were analysed using the independent samples *t*‐test. Expression levels and fold changes of target miRNAs (colostrum and plasma) of the study were calculated with 2^−ΔΔCt^ method (Livak and Schmittgen 2001). The relationship among target miRNAs, SCS and compositional parameters in colostrum was established using Spearman correlation coefficient. The correlations were visualized as a heatmap correlogram. The receiver operator characteristics (ROC) analysis was used to determine a predictive threshold with SCS, compositional parameters and targeted miRNAs for differentiation of the IgG status (low and high). ROC curves for detection of IgG status were obtained for the variables. Sensitivity, specificity, 95% confidence interval and area under the curve (AUC) were calculated. Statistical significance was considered *p* < 0.05.

## 3 Results

**FIGURE 1:**
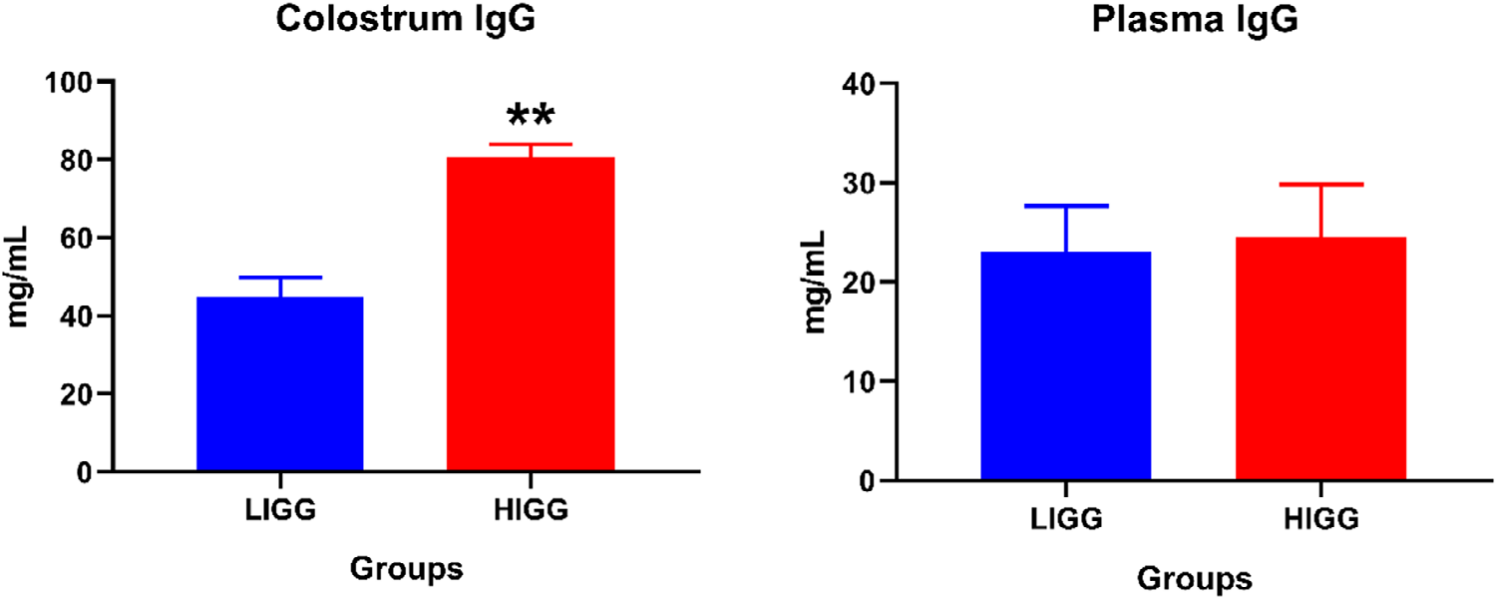
IgG levels in colostrum and plasma samples of LIGG (low IgG) and HIGG (high IgG) groups, **p < 0.01. IgG, Immunoglobulin G.

In the groups formed on the basis of colostrum IgG levels, as expected, there were significant differences in the colostrum samples in terms of IgG levels (*p* < 0.01). However, plasma IgG levels were similar in both groups (Figure 1).

**FIGURE 2:**
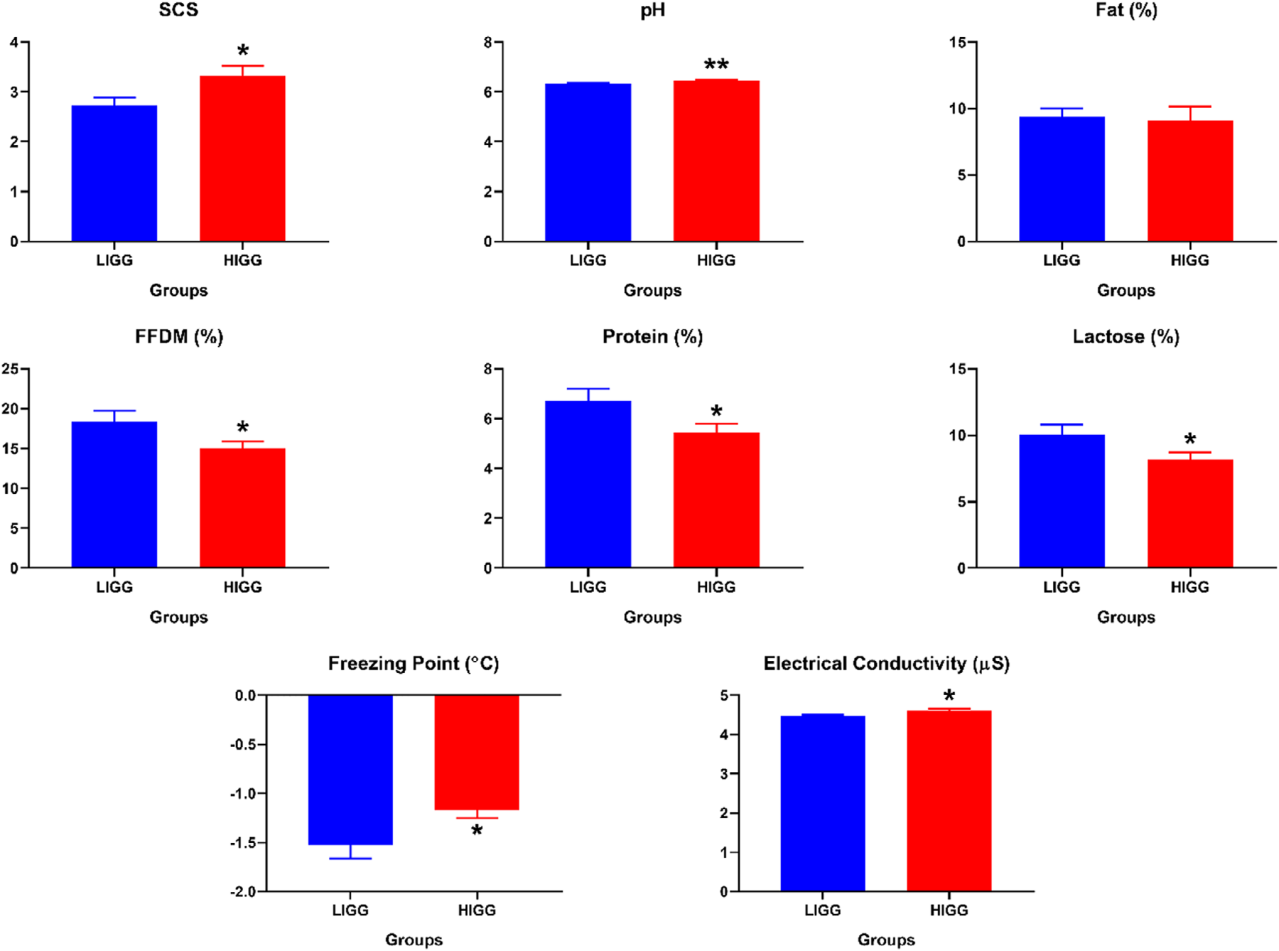
Compositional milk parameters in LIGG (Low IgG) and HIGG (High IgG) groups, *p < 0.05; **p < 0.01. FFDM, fat‐free dry matter; SCS, somatic cell score.

Regarding SCS, it was found that the HIGG group was significantly higher (*p* < 0.05). Additionally, it was determined that the pH, freezing point and EC levels in the HIGG group were significantly higher compared to the LIGG group (*p* < 0.01; *p* < 0.05, respectively). However, among the parameters comprising milk composition, FFDM, protein and lactose levels were found to be lower in the HIGG group (*p* < 0.05) (Figure 2).

**FIGURE 3:**
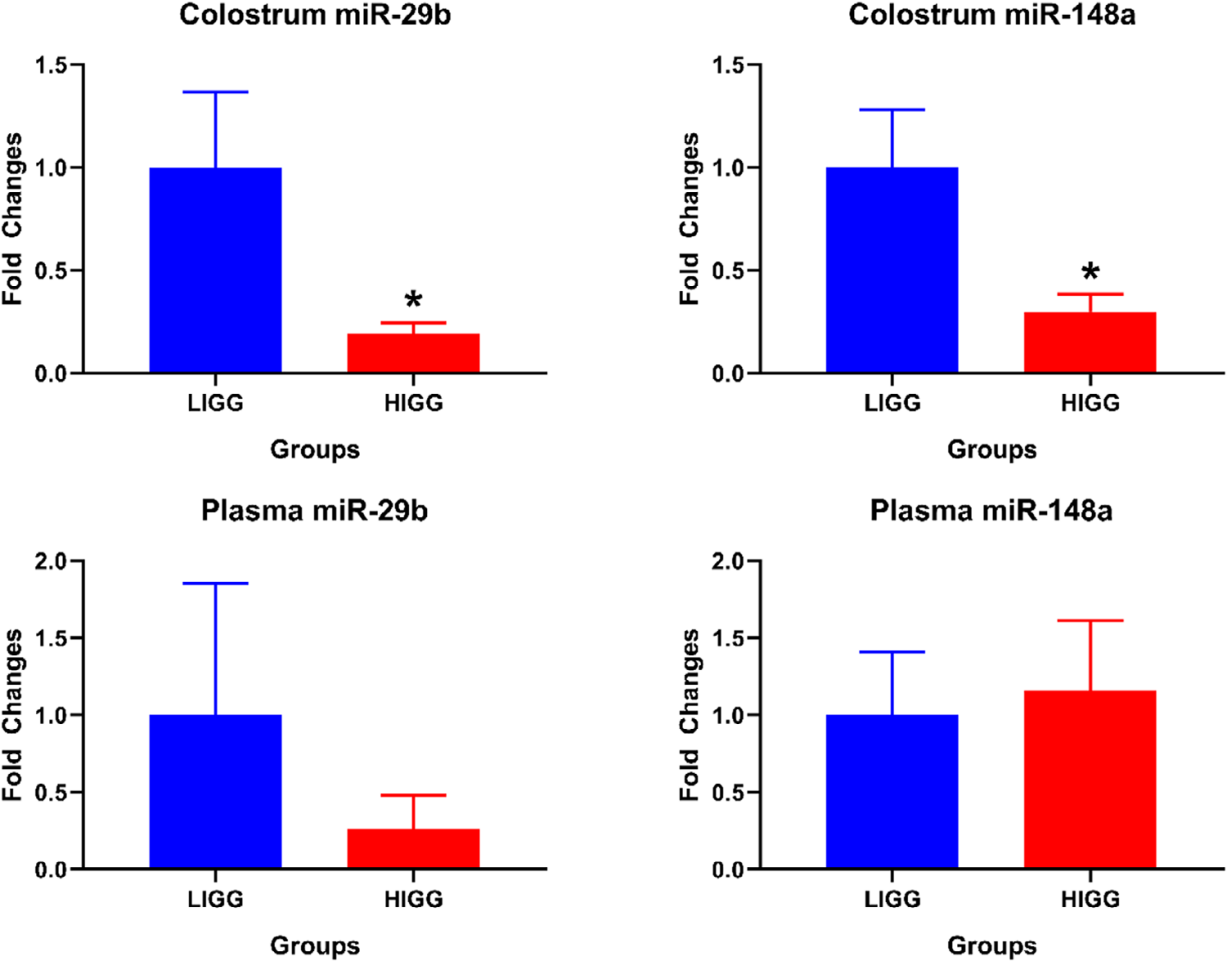
Expression changes of miR‐29b and miR‐148a in colostrum and plasma samples of groups. **LIGG,** low IgG group, **HIGG,** high IgG group. *p < 0.05.

In comparison to the LIGG group, miR‐29b was downregulated approximately 5‐fold in the HIGG group, whereas miR‐148a was downregulated more than 3‐fold in colostrum (*p* < 0.05). Nevertheless, it was observed that the plasma expression levels of these miRNAs showed similar patterns in both the LIGG and HIGG groups (Figure 3).

**FIGURE 4:**
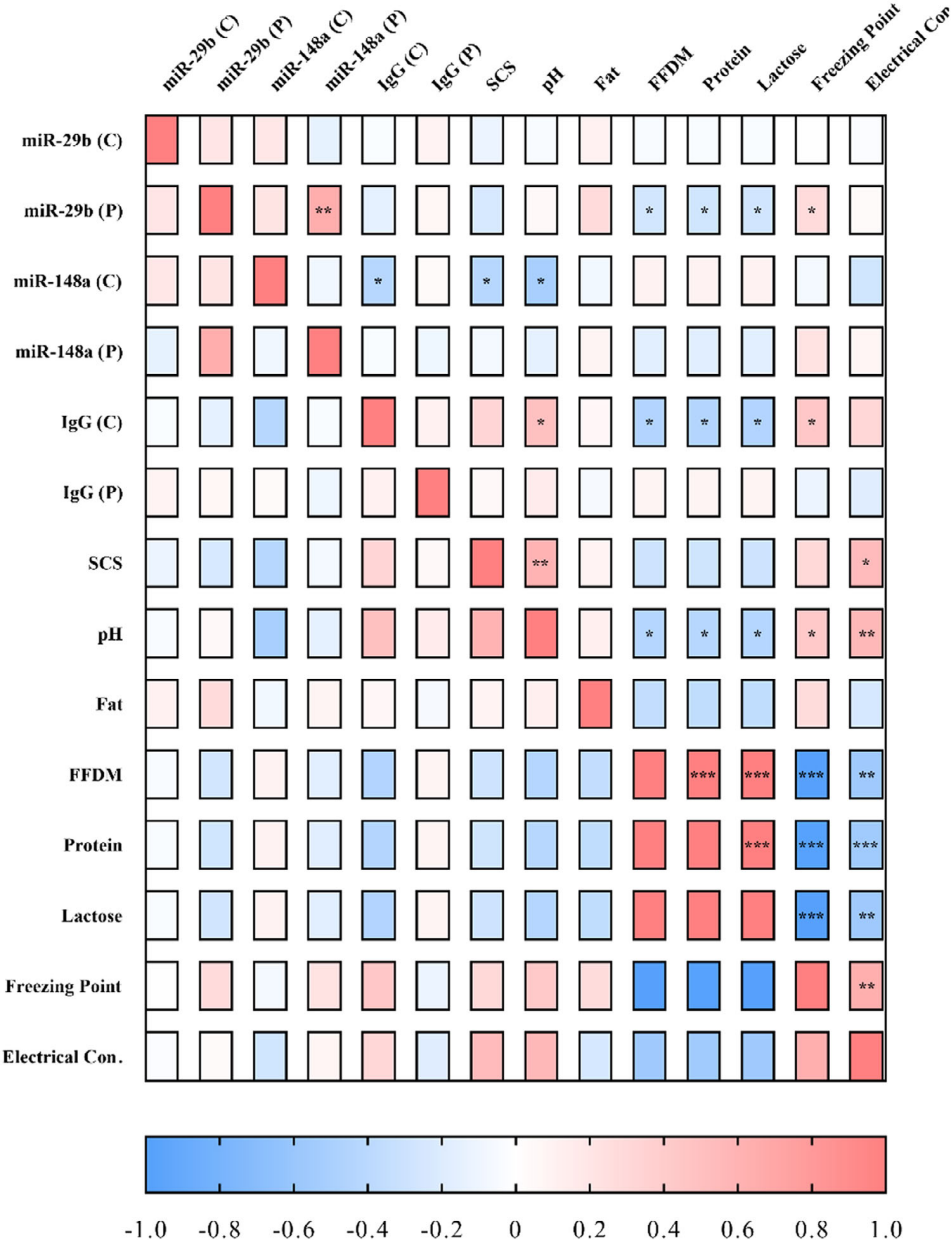
Correlation between miRNAs and quality parameters. FFDM, fat‐free dry matter; IgG, Immunoglobulin G; SCS, somatic cell score.

The study identified significant correlations among the parameters measured in the groups. A positive correlation was found between plasma miR‐29b and miR‐148a (*r*: 0.692, *p* < 0.01), whereas there was a negative correlation between plasma miR‐29b levels and colostrum FFDM (*r*: −0.424, *p* < 0.05), protein (*r*: −0.432, *p* < 0.05) and lactose (*r*: −0.429, *p* < 0.05) parameters and a positive correlation with the freezing point (*r*: 0.428, *p* < 0.05). Additionally, there were negative correlations between colostrum miR‐148a levels and colostrum IgG (*r*: −0.423, *p* < 0.05), SCS (*r*: −0.422, *p* < 0.05) and pH (*r*: −0.507, *p* < 0.05) parameters. However, it was determined that colostrum IgG levels were positively correlated with pH (*r*: 0.493, *p* < 0.05) and freezing point (*r*: 0.444, *p* < 0.05) and negatively correlated with FFDM (*r*: −0.457, *p* < 0.05), protein (*r*: −0.451, *p* < 0.05) and lactose (*r*: −0.453, *p* < 0.05). A positive correlation was found between SCS and pH (*r*: 0.584, *p* < 0.01). In addition, there was a negative correlation between pH and FFDM (*r*: −0.436, *p* < 0.05), protein (*r*: −0.428, *p* < 0.05) and lactose (*r*: −0.436, *p* < 0.05) and a positive correlation with freezing point (*r*: 0.423, *p* < 0.05) and EC (*r*: 0.556, *p* < 0.01). Moreover, FFDM was correlated with protein (0.996, *p* < 0.001), lactose (*r*: 0.993, *p* < 0.001), freezing point (*r*: −0.990, *p* < 0.001) and EC (*r*: −0.562, *p* < 0.01). Although protein was correlated with lactose (*r*: 0.994, *p* < 0.001), freezing point (*r*: −0.989, *p* < 0.001) and EC (*r*: −0.556, *p* < 0.001), lactose was negatively correlated with freezing point (*r*: −0.990, *p* < 0.001) and EC (*r*: −0.562, *p* < 0.01). Finally, there was also a positive correlation between freezing point and EC (*r*: 0.622, *p* < 0.01) (Figure 4).

**FIGURE 5:**
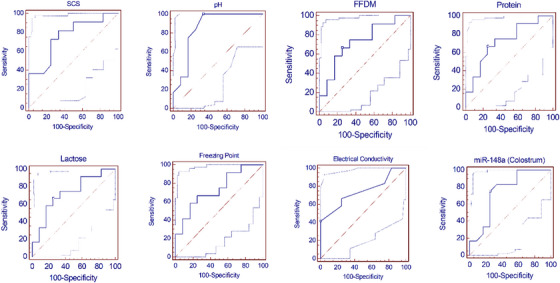
ROC curve for compositional parameters in colostrum between LIGG (Low IgG) and HIGG (High IgG) groups. FFDM, fat‐free dry matter; SCS, somatic cell score.

ROC curves and the AUC values through LIGG and HIGG groups are presented in Figure 5. The cut‐off values of SCS, pH, FFDM, protein, lactose, freezing point, EC and miR‐148a (colostrum) were found to be >2.66, >6.31, ≤15.2, ≤5.5, ≤8.3, >−1.17, >4.6 and ≤0.33, respectively, in HIGG and LIGG groups. All AUC values of these parameters were determined statistically significant (*p* < 0.05). Additionally, the sensitivity and specificity of pH were 100% and 66.7%, of SCS were 81.8% and 66.7% and of miR‐148a (Colostrum) were 75% and 75% in HIGG and LIGG groups. The sensitivity and specificity values of other parameters are given in Table 2.

**TABLE 2:** Receiver operator characteristics (ROC) curve analysis for determination of the cut‐off values as predictor of the Immunoglobulin G (IgG) status between LIGG (Low IgG) and HIGG (High IgG) groups.

Parameters	Threshold	Se	95% CL for Se	Sp	95% CL for Sp	AUC	*p*
**SCS**	>2.66	81.80	48.20–97.20	66.70	34.90–89.90	0.761	0.011
**Ph**	>6.31	100.00	73.40–100.0	66.70	34.90–89.90	0.854	<0.001
**FFDM**	≤15.20	66.70	34.90–89.90	75.00	42.80–94.20	0.729	0.029
**Protein**	≤5.50	66.70	34.90–89.90	75.00	42.80–94.20	0.726	0.032
**Lactose**	≤8.30	66.70	34.90–89.90	75.00	42.80–94.20	0.729	0.029
**FP**	>‐1.17	58.30	27.80–84.70	83.30	51.60–97.40	0.736	0.023
**EC**	>4.60	41.70	15.30–72.20	100.00	73.40–100.0	0.743	0.018
**miR‐148 (C)**	≤0.33	75.00	42.80–94.20	75.00	42.80–94.20	0.750	0.014

Abbreviations: AUC, area under the curve; C, colostrum; EC, electrical conductivity; FFDM, fat‐free dry matter; FP, freezing point; SCS, somatic cell score; Se, sensitivity; Sp, specificity.

**FIGURE 6:**
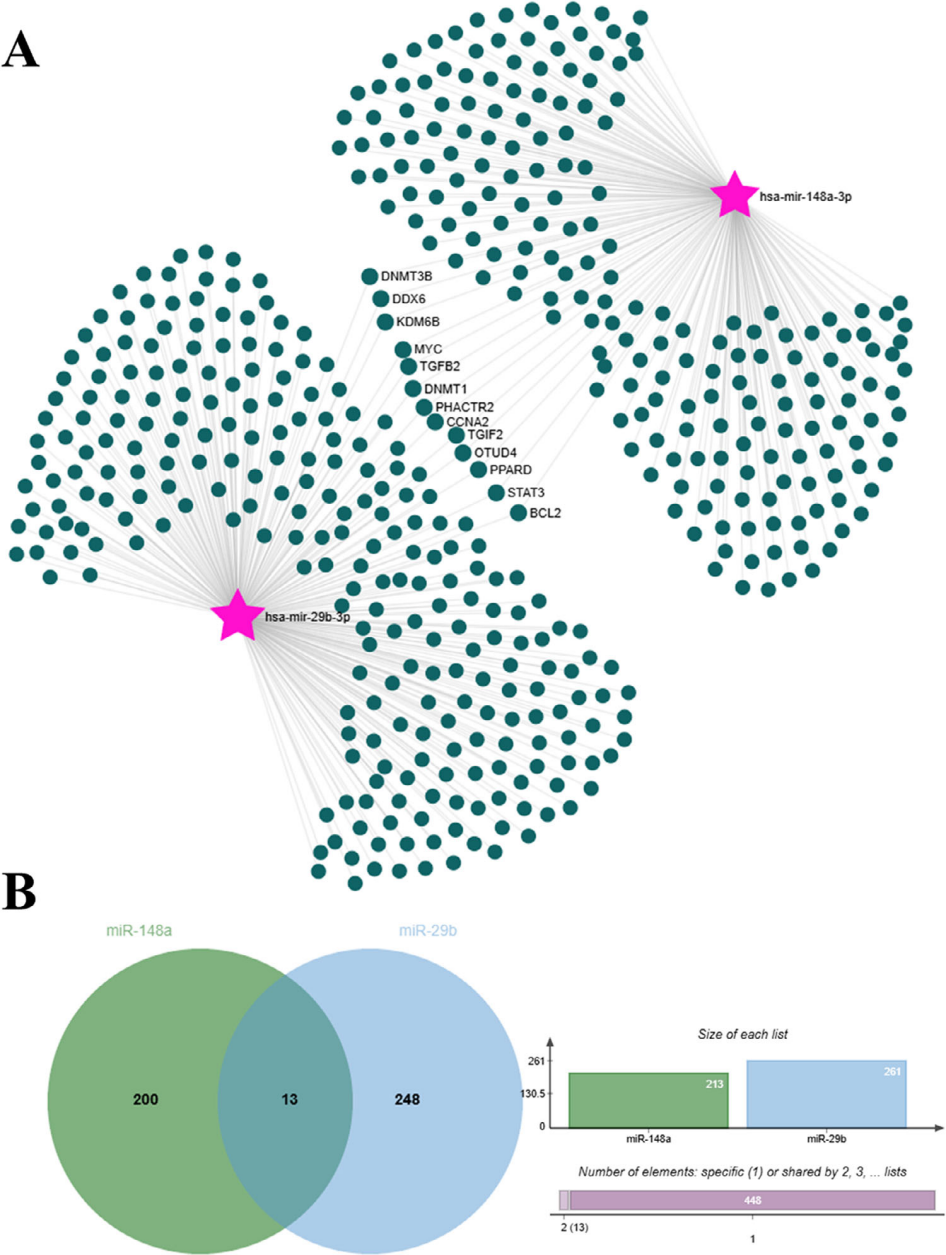
Target genes identified by miRNet databases and a Venn diagram showing overlapping genes for miR‐29b and miR‐148a. (A) Target genes of miR‐29b and miR‐148a identified using the miRNet database. The network visualization shows miRNAs (pink stars) and their predicted target genes (green dots). (B) Venn diagram illustrating the overlap of target genes between miR‐29b and miR‐148a. The green and blue circles represent the unique target genes of miR‐148a and miR‐29b, respectively, while the overlapping region indicates shared target genes. The bar chart provides a numerical representation of unique and shared target genes.

The genes targeted by DEMs are visualized in Figure 6. On the basis of the analyses, it was determined that both miRNAs target a total of 448 genes, as identified using the miRNet and miRTarBase databases. These target genes were supported by experimental data from the miRTarBase and miRNet databases, and the methods used for their identification (e.g., qPCR, luciferase reporter assay and other molecular biology techniques) are detailed in the Supporting Information section. Among the targeted genes, 261 were targeted by miR‐29b, whereas 213 were targeted by miR‐148a. Thirteen genes (*DNMT3B*, *DDX6*, *KDM6B*, *MYC*, *TGFB2*, *DNMT1*, *PHACTR2*, *CCNA2*, *TGIF2*, *OTUD4*, *PPARD*, *STAT3* and *BCL2*) were commonly targeted by both miRNAs.

**FIGURE 7:**
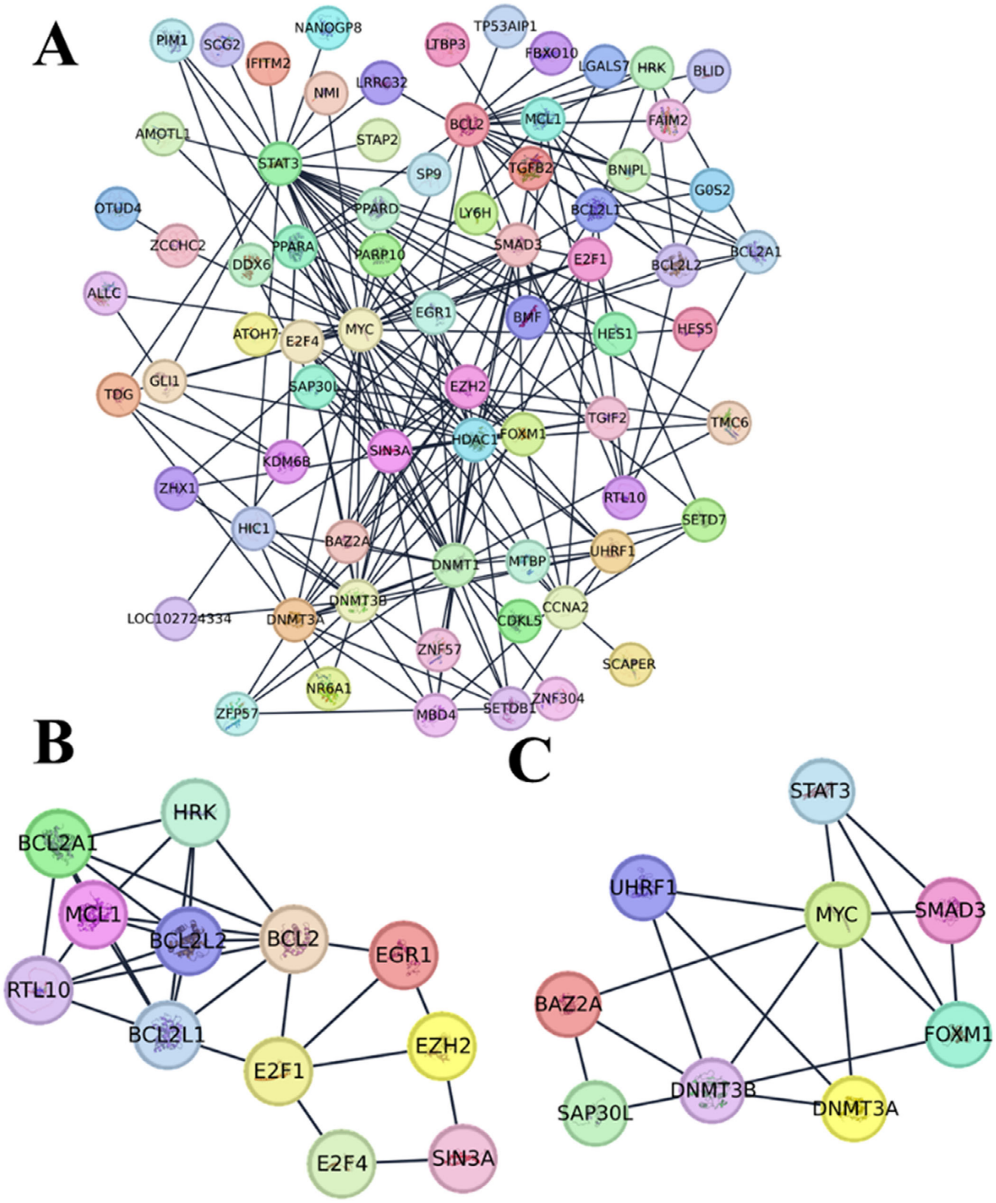
Protein–protein interaction network analysis of targeted genes with MCODE clustering: (A) PPI network analysis of targeted genes; (B) MCODE clustering (score: 5.273) revealing a highly interconnected gene module; (C) MCODE clustering (score: 4.250) highlighting another distinct gene module.

The interactions among the 13 genes targeted by both miRNAs were enriched using the STRING database, and the visualization obtained through Cytoscape analysis is presented in Figure 7. The findings revealed that these 13 genes are part of a network comprising 72 nodes and 221 edges (Figure 7A). Additionally, strong interactions within this network were analysed using the MCODE plugin, and the genes with the highest interaction scores are shown in Figure 7B,C. MCODE analysis identified apoptotic genes, such as *BCL2*, *BCL2L1* and *BCL2L2*, as well as tumour suppressor E2F family genes involved in cell cycle regulation, with the highest interaction score (MCODE score: 5.273, Figure 7B). Furthermore, another cluster with an interaction score of 4.250 was identified, including nine genes. Among these, *SMAD3* and *STAT3*, which are critical for cellular signalling regulation, along with DNMT3 family genes involved in epigenetic modification, were found to exhibit statistically significant interactions (Figure 7C).

**FIGURE 8:**
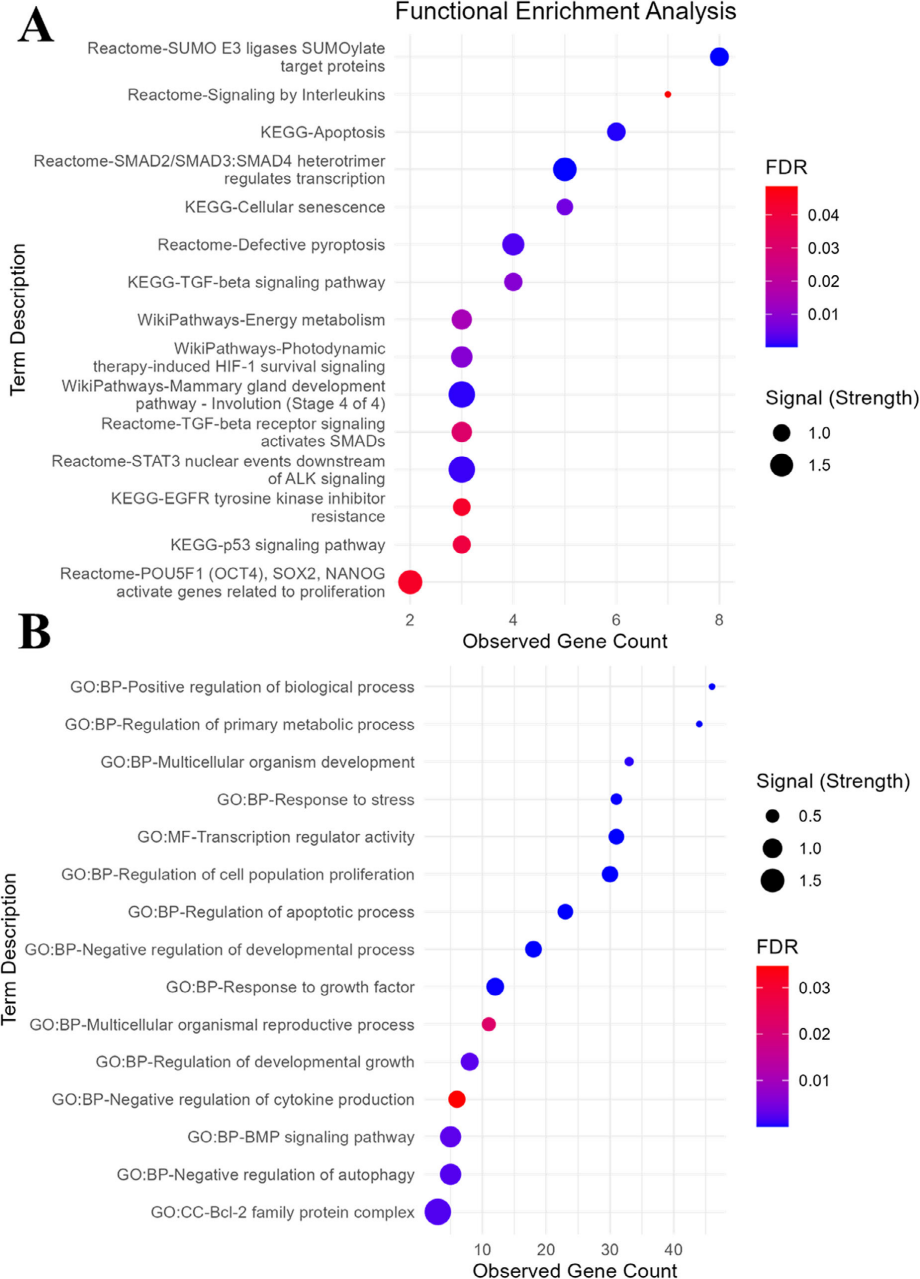
Functional enrichment analysis of targeted genes: (A) the enrichment of targeted genes in biological pathways derived from KEGG, Reactome and WikiPathways databases; (B) Gene Ontology (GO) enrichment analysis for targeted genes, including biological process (BP), molecular function (MF) and cellular component (CC) categories. FDR, false discovery rate.

Functional enrichment analysis was conducted to identify the BPs and pathways regulated by the target genes. The results demonstrated significant involvement of these genes in pathways directly associated with pregnancy, including mammary gland development, energy metabolism and EGFR signalling, alongside critical defence mechanisms, such as apoptosis, pyroptosis, interleukin signalling and the p53 pathway (Figure 8A). Additionally, these genes were found to play pivotal roles in regulating biological and metabolic processes fundamental to growth, development and reproduction. GO analyses further categorized these roles into MF, CC and BP, highlighting the diverse functional capacities of the target genes (Figure 8B).

## 4 Discussion

In mammals, the concentration of IgG in colostrum has emerged as the most critical parameter for determining colostrum quality. Although the IgG levels in colostrum varied among the groups in this study, the plasma levels of this immunoglobulin were found to be similar. Colostrum synthesis is not an instantaneous event but rather a physiological process. It is known that colostrum synthesis begins in the late stages of pregnancy, with maternal immune components like IgG transferring from the blood to the mammary tissue and colostrum (Castro et al. 2011). Consequently, the discrepancy between the IgG levels in colostrum, which is synthesized and accumulated over a specific period in the mammary gland, and the plasma IgG levels are considered an anticipated finding. Furthermore, the findings of this study support the notion that, due to the synepitheliochorial placental structure in ruminants, the IgG necessary for the offspring's immune system is provided postnatally through colostrum rather than via the bloodstream, resulting in lower maternal circulating IgG levels compared to colostrum (Castro et al. 2011).

In sheep, factors such as breed lead to a wide distribution of colostrum IgG levels. The colostrum IgG concentrations in sheep breeds like Blackface, Suffolk and Rasa Aragonesa have been reported to range between 37 and 79 mg/mL (Kessler et al. 2019). In this study, conducted on Awassi sheep, colostrum IgG levels similar to those reported in the literature were found (44.83 ± 4.97 mg/mL in LIGG, 80.71 ± 3.31 mg/mL in HIGG). Significant differences in SCS, pH and compositional parameters in the HIGG were also determined. The high SCS levels in colostrum, which can be physiologically high, are considered noteworthy, especially in the HIGG group. Contrary to findings in sheep, an inverse relationship between SCC and IgG levels in colostrum has been reported in cattle (Puppel et al. 2020). Although no direct significant correlation was found between SCS and colostrum IgG levels in our correlation analyses, the high somatic cell count in colostrum can likely be attributed to the apocrine secretion system (Paape et al. 2001; Özkan et al. 2024). Similarly, pH levels were found to be higher in the HIGG group, with a significant positive correlation between SCS and pH. The positive correlation between colostrum IgG levels and pH indicates a positive relationship among IgG, pH and SCS in colostrum. The positive correlation of EC with SCS and pH is also considered an expected finding (Yakan et al. 2021).

Although a traditional relationship exists between IgG and colostrum quality, the compositional parameters of colostrum, which play a crucial role in meeting the basic metabolic energy needs of the offspring, are also prominent in evaluating colostrum quality (Puppel et al. 2020). In FFDM, protein and lactose levels were found to be significantly lower in the HIGG group. The study conducted by Kessler et al. (2019) indicated significant variation in compositional parameters in colostrum among different sheep breeds, and the findings in this study conducted on Awassi sheep were consistent with another study on the same breed (Higaki et al. 2013). Although studies have been conducted on cattle colostrum, the number of studies investigating the relationship between IgG and these parameters in sheep colostrum is limited (Løkke et al. 2016; Navarro et al. 2018; Todaro et al. 2023). Even if a study in sheep reported a weak positive correlation between IgG and total protein in colostrum, this study found a weak negative correlation between these parameters (Navarro et al. 2015). This discrepancy may be attributed to the fact that the animals included in the study were first‐time parturients, and the processes of colostrogenesis and mammogenesis, as well as other proteins such as α‐lactalbumin and β‐lactalbumin, which significantly influence the total protein content in colostrum, could have contributed to the obtained results (Ding et al. 2011; Galán‐Malo et al. 2014; Fleming et al. 2016; Levieux et al. 2002).

The study found that although miR‐29b and miR‐148a exhibited similar expression patterns in plasma among the groups, both miRNAs were significantly downregulated in colostrum. miR‐29b, a member of the miR‐29 family, is a crucial non‐coding RNA molecule that regulates the expression of genes related to various cellular functions (Xiao et al. 2013). Studies have reported that miR‐29b regulates cell proliferation and apoptosis (Huang et al. 2013; Li et al. 2013). It has been reported that miR‐29b inhibits the growth of the small intestine mucosa through genes such as cyclin‐dependent kinase 2 (CDK2) (Xiao et al. 2013). Therefore, the significant downregulation of this miRNA in the HIGG group might be due to its reduced presence to promote digestive system development in addition to IgG‐related immune development in lambs. Although the plasma levels of this miRNA were similar across the groups, its positive correlation with miR‐148a and freezing point and negative correlation with FFDM, protein and lactose suggest a complex molecular role in colostrum for the development of the offspring. miR‐29b, also thought to be related to energy metabolism, is reported to play a role in the conversion of glucose to lipids in carbohydrate metabolism, which can affect milk quality (Yang et al. 2016). The negative correlation with milk quality parameters, such as FFDM, protein and especially lactose, might stem from this relationship, suggesting that it could be an alternative biomarker in evaluating colostrum quality.

miR‐148a, one of the most highly expressed miRNAs in ruminant milk (Yun et al. 2021), was found to be downregulated in the HIGG group and negatively correlated with IgG, SCS and pH. A study demonstrated that miR‐148a activity in milk protects developing offspring from cancer and similar diseases, and it was reported that miR‐148a levels are downregulated in leukaemia (Golan‐Gerstl et al. 2017). Another study indicated that miR‐148a levels increase in autoimmune diseases (Gonzalez‐Martin et al. 2016). miR‐148a, which is considered to be associated with immunoglobulins, appears to play an active role in immunity and might be suppressed by the HIGG content in colostrum. A recent study reported that miR‐148a in human milk is hydrolysed by IgG (Kompaneets et al. 2022). The correlation between colostrum miR‐148a levels and colostrum IgG levels in this study supports this finding. Additionally, miR‐148a was found to be negatively correlated with SCS and pH in this study. This miRNA, which actively regulates the immune system and is negatively correlated with IgG, might be associated with somatic cells consisting of mammary epithelial cells and leukocytes, suggesting its potential as a candidate for determining colostrum quality in sheep.

The similar expression patterns of the studied miRNAs in maternal plasma, contrasted with their differential expression levels in colostrum associated with IgG, suggest that the activity of these miRNAs may be linked to local molecular activity, such as in the mammary gland. On the other hand, bioinformatics analyses revealed that both miRNAs investigated in this study target over 200 genes, among which *DNMT3B*, *DDX6*, *KDM6B*, *MYC*, *TGFB2*, *DNMT1*, *PHACTR2*, *CCNA2*, *TGIF2*, *OTUD4*, *PPARD*, *STAT3* and *BCL2* were identified as common targets. Changes in these genes are associated not only with epigenetic regulation but also with the control of cellular proliferation and apoptosis mechanisms, immune system function, cellular differentiation and development and metabolic regulation (Park et al. 2021; Lei et al. 2022; Srivastava et al. 2023). These findings provide significant insights into the activity of these miRNAs in high‐IgG colostrum, considered high‐quality, which is essential for both maternal health and the development of colostrum‐fed offspring. Functional enrichment analysis has demonstrated that miR‐29b and miR‐148a play critical roles not only in BPs such as mammary gland development and HIF‐1 regulation but also in the modulation of key signalling pathways, including interleukins, apoptosis, TGF‐β, STAT3 and p53. These signalling pathways are reported in the literature to be essential for the healthy progression of pregnancy and the regulation of immune responses during the postpartum period (Ali Harby et al. 2022; Duan et al. 2021; Li et al. 2024; Sen et al. 2024). Collectively, these findings underscore the multifaceted regulatory functions of the target genes, contributing to the coordination and maintenance of various cellular and BPs.

## 5 Conclusion

This study is the first to examine the changes in miR‐29b and miR‐148a, which show different patterns in colostrum based on IgG levels, and to investigate the relationships between these miRNAs and compositional parameters. miR‐29b, suggested as a therapeutic target in treating intestinal disorders, might be an important molecular target for lamb development, whereas miR‐148a could be a potential biomarker for colostrum quality due to its relationship with IgG. Moreover, the present study showed that SCS and pH might be useful diagnostic parameters for IgG status of colostrum because of higher sensitivity rates and AUC values. Further studies involving a larger number of animals under similar principles are recommended to better understand the molecular mechanisms of immunoregulation during colostrogenesis and lactation in sheep, particularly by examining the relationships between these miRNAs and IgG in both colostrum and mature milk.

## Author Contributions

**Hüseyin Özkan**: formal analysis, investigation, methodology, writing – original draft. **Ufuk Kaya**: data curation, methodology, formal analysis, writing – review and editing. **Hasan Hüseyin Keçeli**: methodology, writing – review and editing. **Ramazan Sertkol**: methodology. **Mustafa Kemal Saribay**: methodology, writing – review and editing.

## Ethics Statement

The research was approved by the Local Animal Experiments Ethics Committee of Hatay Mustafa Kemal University (Decision no.: 2022/05‐12).

## Conflicts of Interest

The authors declare no conflicts of interest.

## Supporting information



Supporting Information

## Data Availability

The datasets generated during and/or analysed during the current study are available from the corresponding author on reasonable request.
